# Supplementary material for: Genetic Variants Identified by Whole Exome Sequencing in a Large Italian Family with High Plasma Levels of Factor VIII and Von Willebrand Factor
Source: Int J Mol Sci. 2023 Sep 15;24(18):14167. doi: 10.3390/ijms241814167 (PMC10532311; doi:10.3390/ijms241814167)
Supplement: Supplementary file 1 [file ijms-24-14167-s001.zip › ijms-2502887-supplementary.pdf]

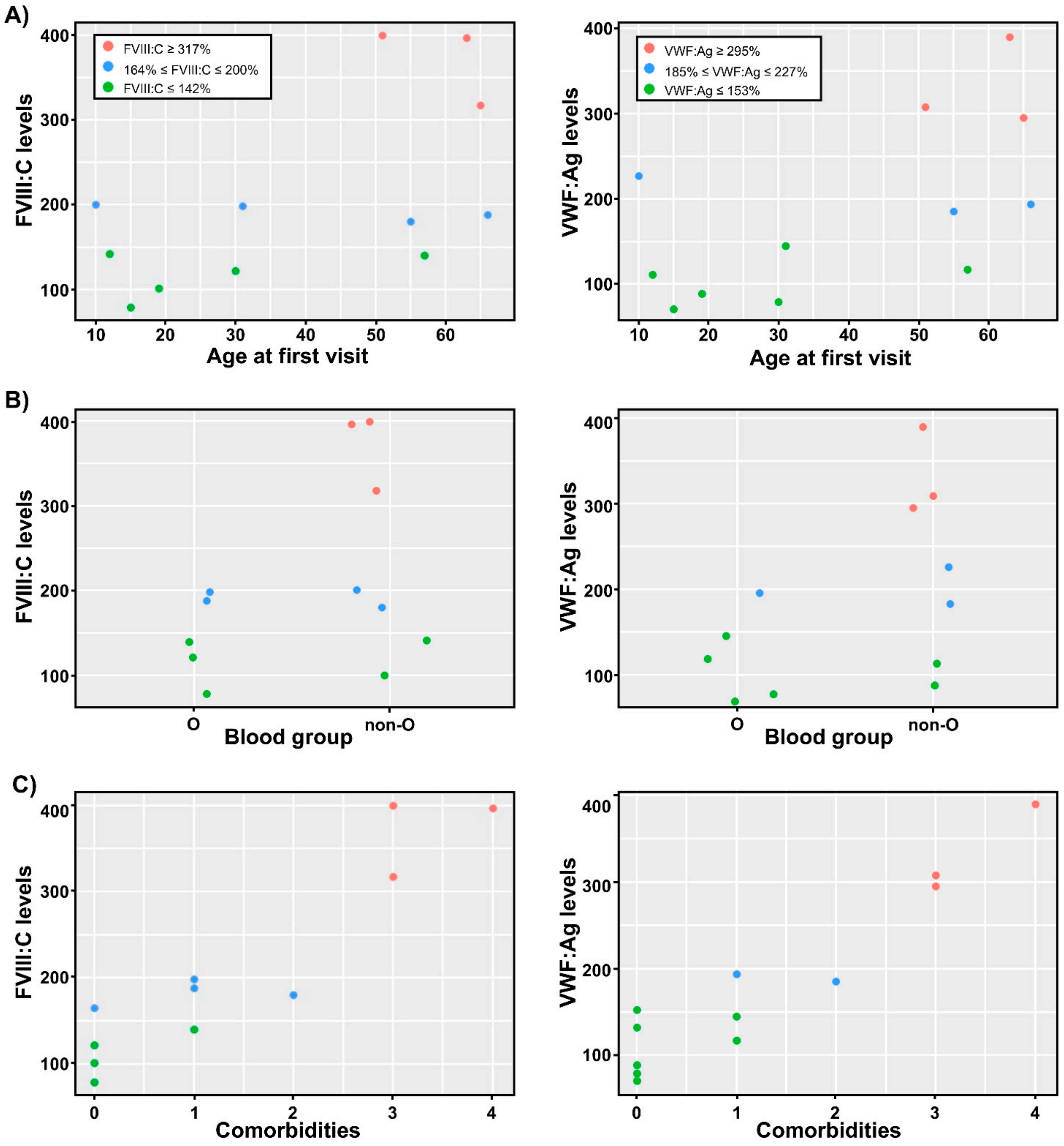

Figure S1: Dot plots of family members showing the distribution of FVIII:C and VWF:Ag levels (%; y-axis) and **(A)** age at first visit, **(B)** blood group, **(C)** number of comorbidities (i.e. obesity, overweight, diabetes, dyslipidemia, hypertension, tumor) (x-axis).

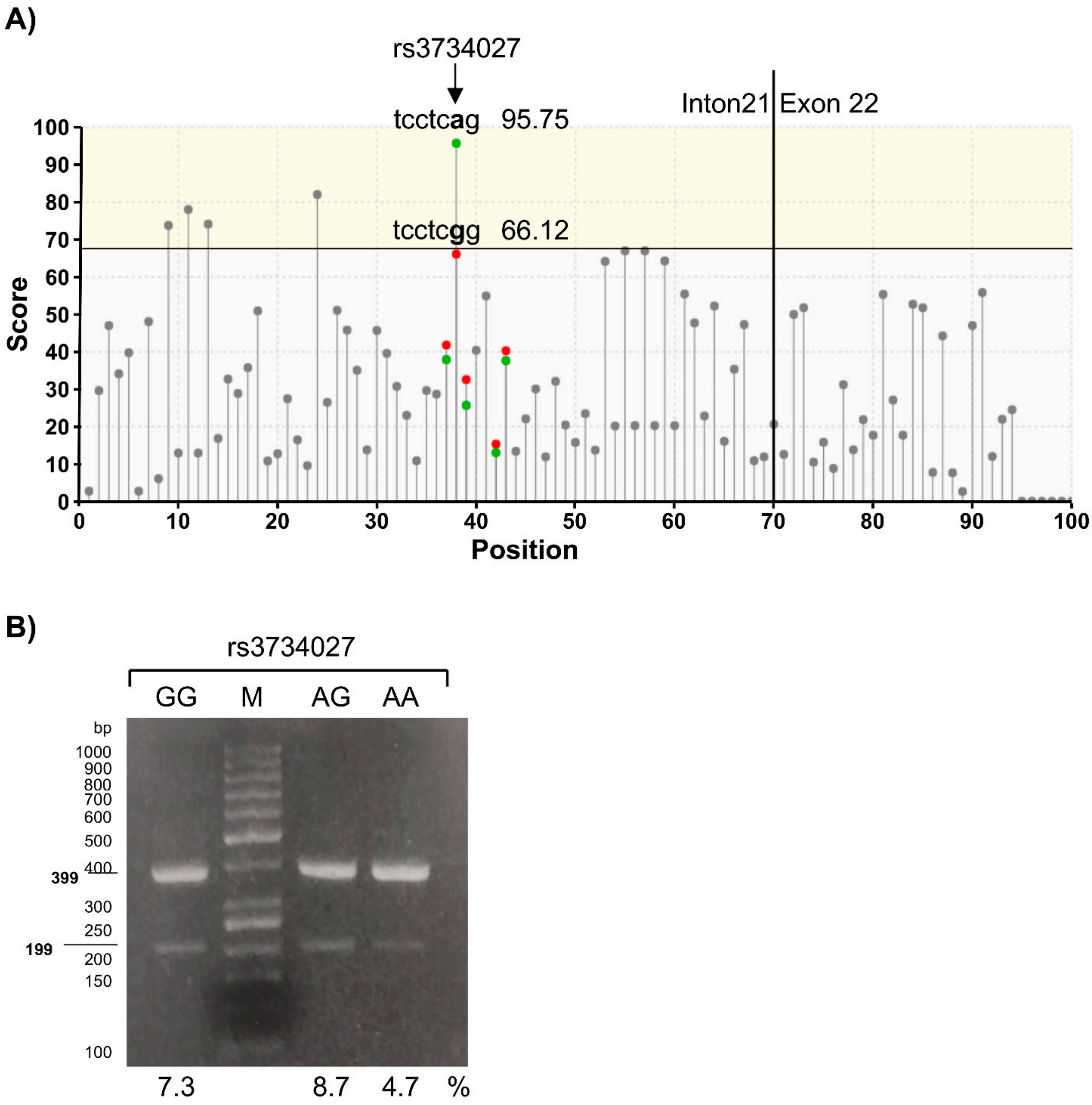

Figure S2: Analysis of rs3734027 (A>G) variant in *CYFIP2*. **(A)** Human Splicing Finder output shows the branch point score (y-axis) and the nucleotide position (x-axis). Values above the threshold are in the yellow area. The rs3734027 variant is indicated by an arrow. The scores of the reference sequence and the variant sequence are indicated by green and red circles, respectively. **(B)** Agarose gel electrophoresis shows *CYFIP2* cDNA bands amplified from heterozygous (AG) and homozygous (AA and GG) individuals. The percentage of the skipped isoform is reported below the gel.

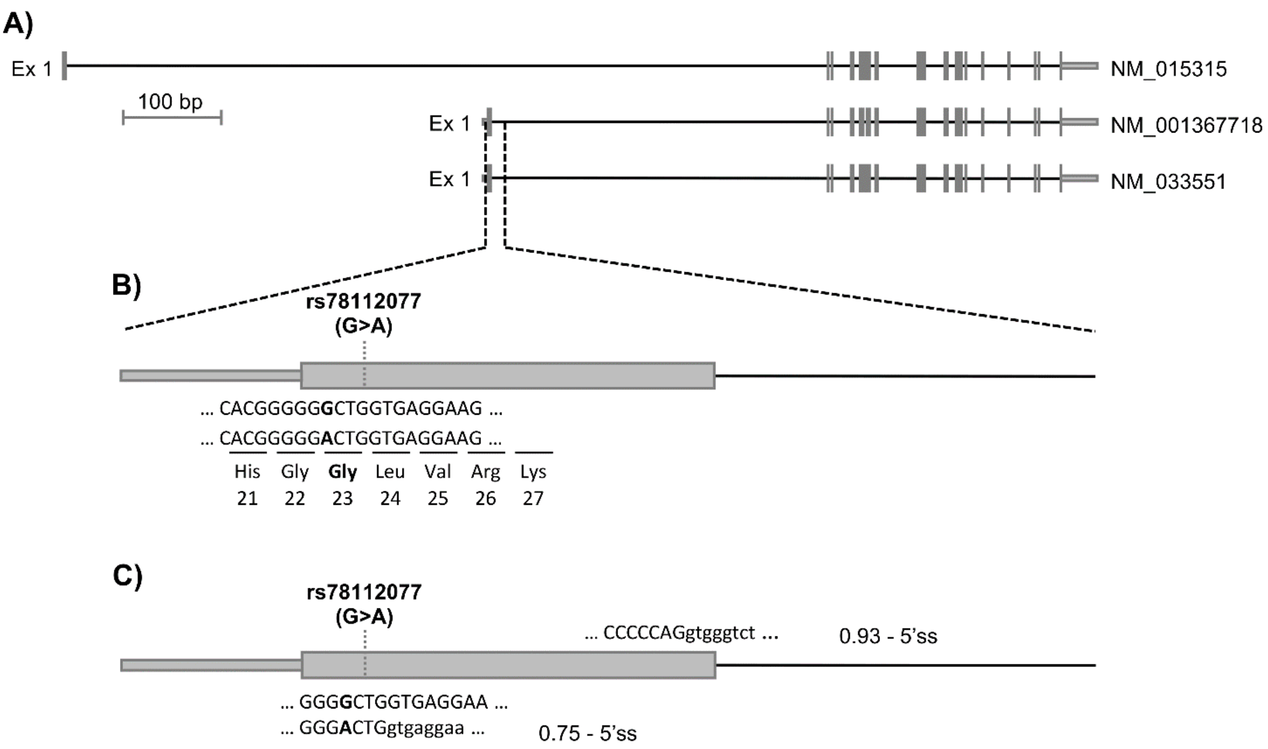

Figure S3: Analysis of rs78112077 (G>A) variant in *LARP1*. Variant localization in *LARP1* transcripts **(A)** and possible effect on codon translation **(B)** and splicing **(C)**.

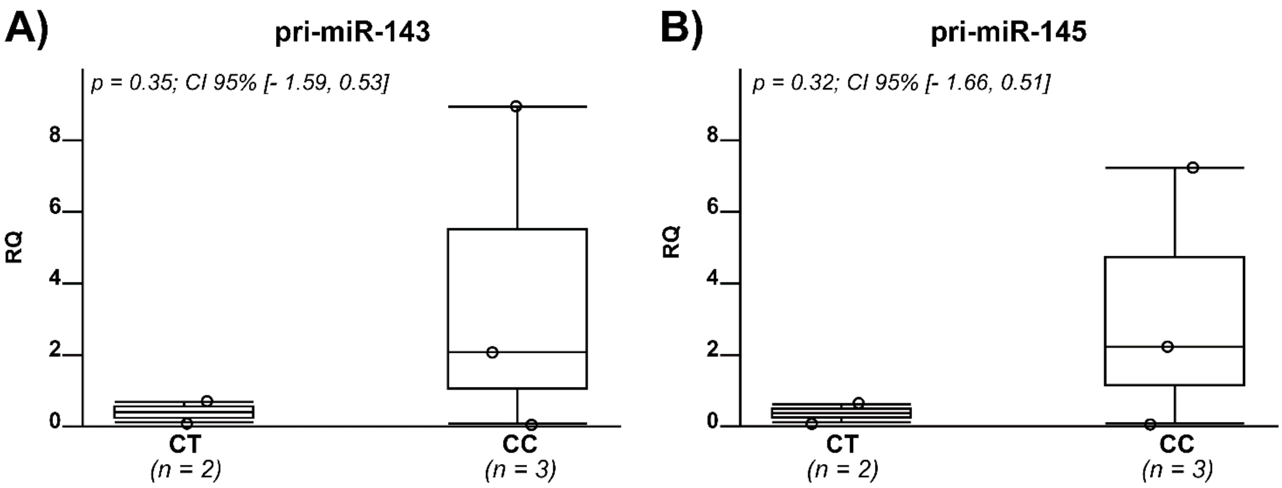

Figure S4: rs13158382 (C>T) variant and transcript levels in heterozygous and homozygous family members. Box plots show the relative quantitation (RQ) of **(A)** pri-miR-143 and **(B)** pri-miR-145 in heterozygous (CT) and homozygous (CC) family members. “n” denotes the number of analyzed individuals for each genotype.
